# Resolving Nanoslip, Solvation Inertia, and Charge Dynamics at Vibrating Solid–Liquid Interface

**DOI:** 10.1002/smll.202505067

**Published:** 2025-07-31

**Authors:** Nikhil Bhalla, Yeeun Song, Ju‐Yeon Jo, Doojin Lee, Amir Farokh Payam

**Affiliations:** ^1^ Nanotechnology and Integrated Bioengineering Centre (NIBEC) School of Engineering Ulster University 2‐24 York Street Belfast Northern Ireland BT15 1AP UK; ^2^ School of Polymer Science and Engineering & Department of Polymer Engineering, Graduate School Chonnam National University 77 Yongbong‐ro Buk‐gu Gwangju 61186 Republic of Korea; ^3^ Graduate School of Energy Science Kyoto University Yoshida‐Honmachi, Sakyo‐ku Kyoto 606‐8501 Japan

**Keywords:** AFM, ionic‐slip, ions‐inertia, QCM‐D, solid‐liquid‐interface

## Abstract

When a liquid contacts a charged solid surface, counterions in the liquid accumulate near the interface—a process traditionally described by models such as Helmholtz, Stern, and Debye‐Hückel. However, these frameworks overlook the complex interplay between inertia and surface charge, and they simplify ions as mere point charges. This study employs vibrating solid surfaces to decouple and investigate the effects of inertia, ion‐slipping, and electrostatic interactions at the molecular scale. This approach reveals “inertial layer” in the initial liquid strata, which plays a critical role in governing interface dynamics. Within this layer, a tunable Helmholtz zone is identified, where mechanical stiffness and electrostatic forces adjust in response to ion concentration. Beyond this lies a Debye screening region characterized by repulsive forces and electrostatic decoupling from the double‐layer capacitor model. Using phosphate‐buffered saline (PBS) as a model electrolyte, it is demonstrated that low ionic strength enhances interfacial stability, while high concentrations increase electrostatic repulsion, influencing nanoscale mechanical behavior. These insights refine the understanding of interfacial phenomena and hold significant implications for biosensing, catalysis, and energy storage technologies.

## Graphical abstract

Schematic illustrating solid‐liquid interface characterization: a quartz crystal microbalance with a dissipation mode sensor maps electric potential across inner Helmholtz, outer Helmholtz, and diffuse layers, while an AFM probe resolves hydration and ion layering at a gold surface.

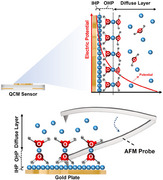

## 1 Introduction

**Figure 1:**
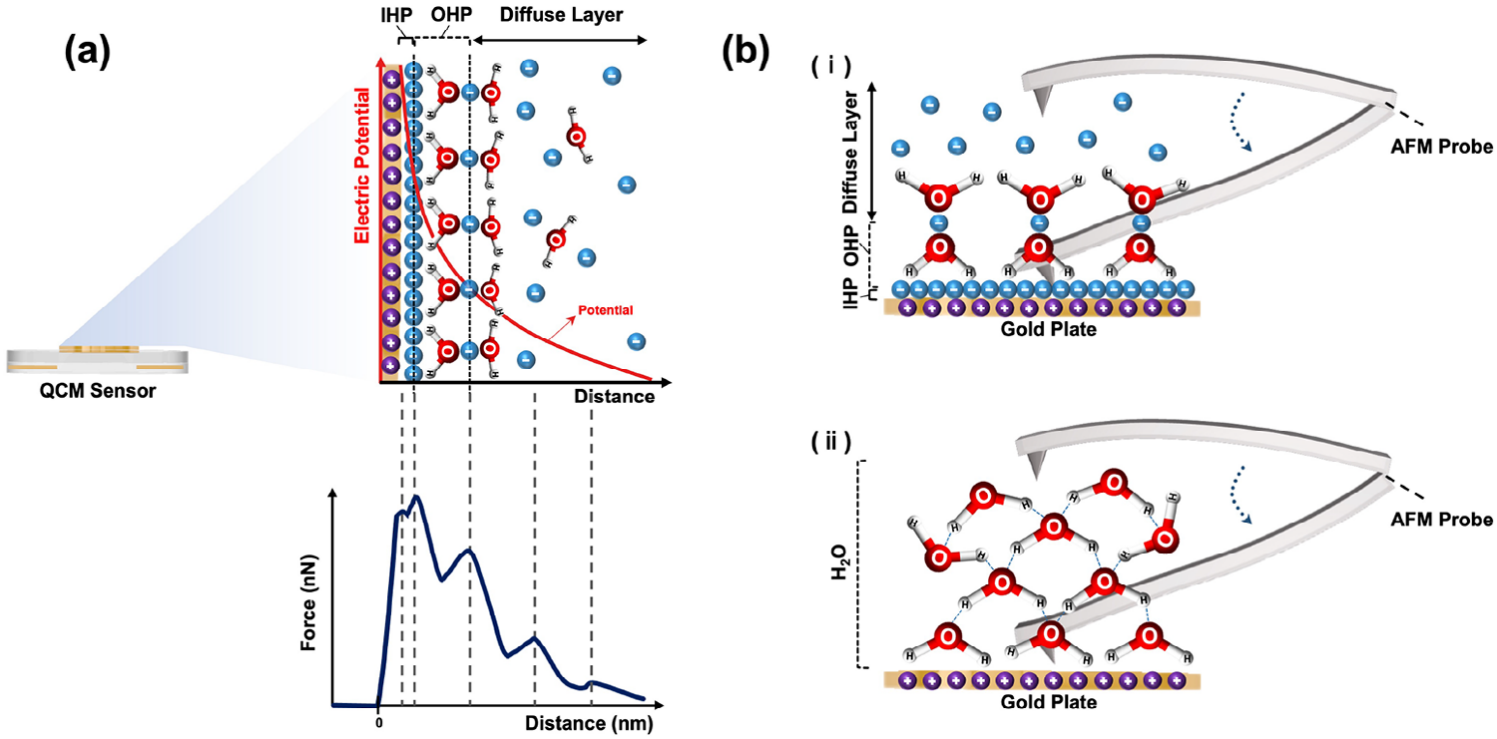
Solid–liquid interface a) depicts the vibrating solid surface of a quartz crystal microbalance (QCM), showcasing its interfacial layers comprising the Inner Helmholtz Plane (IHP) and the Outer Helmholtz Plane (OHP), along with a diffuse layer extending into the bulk liquid. A schematic illustrating typical forces exerted by these layers is also presented, corresponding to their respective distances from the solid surface. b) illustrates the measurement technique employing atomic force microscopy to investigate a liquid solution composed of ions (i) and water (ii), aimed at elucidating the forces depicted in the schematic of part (a).

When an electrically charged vibrating solid comes into contact with a fluid, the free charges in the solution experience a Coulombic force,^[^ ^1^, ^2^, ^3^ ^]^ attracting counterions (ions with opposite charge to the surface) and repelling coions (ions with the same charge as the surface). Typically, this force is inversely proportional to the square of the distance from the solid charged surface,^[^ ^4^ ^]^ causing an exponential decline in the influence of the solid surface's charge on the liquid medium, as shown in **Figure** **1**a. This interaction leads to the formation of an electric double layer at the solid–liquid interface, which includes the Inner Helmholtz Plane (IHP), Outer Helmholtz Plane (OHP), and a diffuse layer (see Figure 1a).^[^ ^5^, ^6^ ^]^ Classical models, such as those proposed by Helmholtz, Stern, Debye–Huckel and Gouy–Chapman–Stern model^[^ ^7^ ^]^ describe this phenomenon by treating ions as point charges in a continuous solvent. However, these models often overlook the precise interplay between inertia and surface charge, as well as specific solvent interactions and nanoscale molecular effects.^[^ ^8^ ^]^

This spatial distribution of ions on a solid surface influences processes across multiple fields, including electrochemistry,^[^ ^9^ ^]^ colloidal science,^[^ ^10^ ^]^ heterogeneous catalysis,^[^ ^11^, ^12^ ^]^ surface science,^[^ ^13^ ^]^ and biology.^[^ ^14^ ^]^ For example, in electrochemistry, where the conversion of chemical energy to electrical energy occurs at the interface between an electrode and an electrolyte solution, the spatial distribution of ions influences reaction kinetics, charge transfer processes, and overall device performance.^[^ ^15^ ^]^ The arrangement of ions near the electrode surface dictates parameters such as double‐layer formation, electrode polarization, and electrochemical reactions, thereby shaping the efficiency and selectivity of electrochemical sensing systems.^[^ ^16^, ^17^ ^]^ Within the domain of colloidal science, understanding the spatial distribution of ions is important in precise control of colloidal stability through mechanisms such as electrostatic repulsion, van der Waals interactions, and steric hindrance, leading to tailored properties of colloidal suspensions.^[^ ^18^, ^19^, ^20^ ^]^ Furthermore, the science of catalysis relies on the spatial arrangement of ions to modulate catalyst activity, selectivity, and stability.^[^ ^21^, ^22^ ^]^ The interaction between ions and catalytic surfaces governs processes such as adsorption, desorption, and surface reaction kinetics, ultimately determining the efficiency and specificity of catalytic transformations in various industrial, environmental, biological, and energy harvesting applications.^[^ ^23^, ^24^, ^25^, ^26^, ^27^, ^28^ ^]^

Despite decades of development in classical electric‐double‐layer theory, key questions remain about how inertia, discrete solvent structure, and finite ion size conspire to control interfacial dynamics at angstrom length‐scales. Resolving these effects is critical not only for fundamental nanoscale electrostatics but also for emerging applications—from ultra‐sensitive biosensors that exploit hydration forces up to a few nanometers, to next‐generation capacitive energy storage devices where ion slippage can limit power density. By directly coupling MHz‐frequency vibration with atomic‐scale force mapping, our work provides the quantitative picture of how an “inertial layer” of solvent and the ordering of its ions take place on the solid surface. These insights open routes to engineer interfacial friction, dissipation, and capacitance in nanofluidic, catalytic, and sensing architectures.

In our investigation, we observe anomalous changes in the vibrational properties of the vibrating solid‐liquid interface when the vibrating solid is exposed to liquid solutions of varied ionic strength. We associated these anomalous changes in the vibration with the formation of an electric double layer and structural changes in the nanoscale hydration/solvation layers on the vibrating solid. Essentially, the water adsorbs weakly on gold (the top surface of our vibrating solid), similar to its binding on bulk ice, stabilized through direct bonding with gold atoms and hydrogen bonding among water molecules.^[^ ^29^, ^30^, ^31^, ^32^ ^]^ Typically, water molecules arrange in a pseudo‐tetrahedral environment at the interface, with uncoordinated protons pointing away from the gold surface.^[^ ^29^, ^33^, ^34^ ^]^ Our findings also reveal that the mass of ions is pivotal in establishing a stable, thin layer of ions before their charge begins to contribute to the solid–liquid interfacial effects. These effects encompass anomalous energy dissipation resulting from the viscoelastic modulation of hydrated ions and the slipping of these hydrated ions within the electrical double layer, which we elucidate in our study. Furthermore, we quantify the forces exerted by the ionic layers within the electrical double layer.

## 2 Results and Discussion

**Figure 2:**
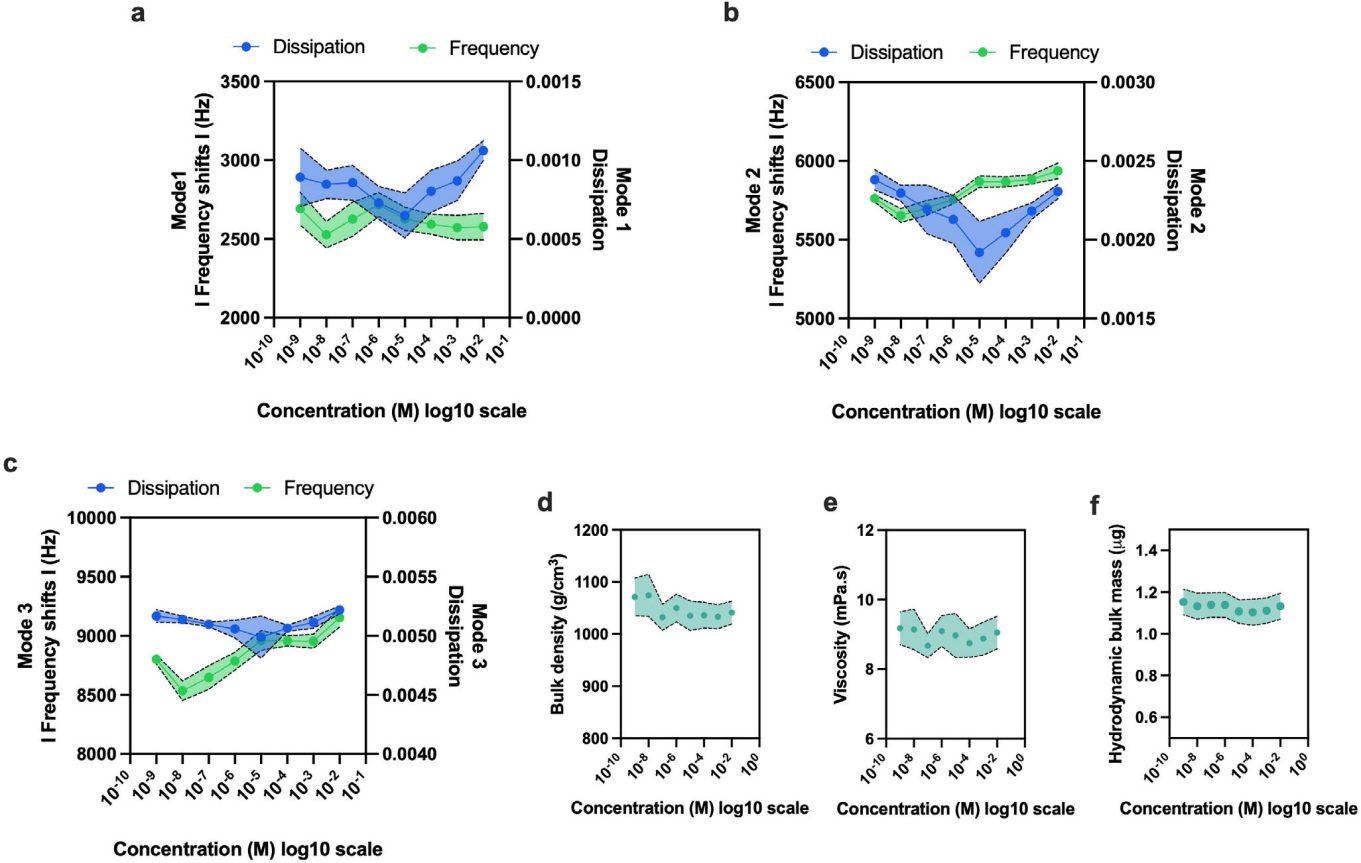
Vibrating solid multi frequency analysis a) frequency and dissipation shifts observed in the vibrating solid at its natural frequency of 10 MHz (mode 1) when exposed to ionic buffer of concentration ranging from 10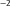 M to 10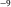 M; b) frequency and dissipation shifts observed in the vibrating solid at its overtone frequency of 30 MHz (mode 2) when exposed to ionic buffer of concentration ranging from 10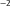 M to 10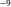 M; c) frequency and dissipation shifts observed in the vibrating solid at its overtone frequency of 50 MHz (mode 3) when exposed to ionic buffer of concentration ranging from 10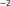 M to 10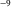 M; d) bulk density, e) bulk viscosity, and f) hydrodynamic bulk mass of the ionic buffer of concentration ranging from 10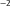 M to 10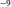 M. Note that the error shaded region in the (a) to (f) plots corresponds to the standard deviation error bands for multiple measurements (n ≥ 3).

We initially examined the impact of ions on the oscillating solid surface by analyzing the energy dissipation and frequency shifts that occur in the vibrating solid during its interaction with the ions. In addition to the monitoring of the natural vibration frequency of the solid at 10 MHz (mode 1 in **Figure** **2**a), we also explore the overtones of the vibrating solid at 30 MHz (mode 2 in Figure 2b) and 50 MHz (mode 3 in Figure 2c), respectively. It is widely recognized that the sensing depth (*D*) decreases as the frequency of the quartz crystal increases (f ∝ 1/D).^[^ ^35^, ^36^ ^]^ Due to this varying sensing range, simultaneous measurements of modes 1, 2, and 3 overtones provide a qualitative effect of the spatially distributed ions interaction with the vibrating solution surface.^[^ ^35^ ^]^

Consequently, compared to mode 1, modes 2 and 3 exhibit lower sensitivity to changes in the bulk, with mode 3 demonstrating the highest sensitivity to fluid layers near the surface of the vibrating solid. From Figure 2a–c, we note a decline in dissipation up to a concentration of 10 μM, followed by an increase thereafter. These dissipation shifts are more pronounced in mode 2 (Figure 2b) as opposed to modes 1 and 3. This can be attributed to the elimination of the bulk effect (influence of ions of the bulk liquid in contrast to layers of liquid closer to the solid surface) seen in mode 1, allowing for a more effective examination of the regions comprising the IHP and OHP in mode 2. In mode 3, the sensing thickness is thinner than in mode 2, where we expect a more stable ionic layer compared to the thickness investigated by mode 2. As a result, there is less variation in the dissipation shifts in mode 3 compared to mode 2.

In our investigation, we observed a concentration‐dependent effect on dissipation at the gold–water interface. Initially, when water molecules approach a gold surface, they can adsorb through physical adsorption involving van der Waals forces or through chemical adsorption, which involves stronger interactions.^[^ ^29^, ^37^, ^38^, ^39^ ^]^ The first layer of water molecules at the gold interface is often well‐ordered due to direct interaction with the gold atoms. These water molecules can form a structured layer where they are more tightly bound than in the bulk phase. Beyond the first hydration layer, the structure becomes more similar to bulk water but still exhibits some order due to the influence of the underlying layers. Due to the weak bonding between water molecules and the gold surface, there is significant mobility or dissipation of water molecules near the gold surface, maintaining a fluid‐like state. As the concentration of ions in the solution increases from 0 to 10 μM PBS, a stable thin layer of ions forms on the surface, known as the IHP and OHP. Essentially, as the stable IHP/OHP forms, the rising concentration of ions in the solution, ranging from 0 to 10 μM, and the introduction of PBS ions stabilizes the electrical double layer (EDL). These ions adsorb onto the gold surface, enhancing the surface charge density and promoting a more organized and stable EDL, amplifying the inertia on the surface, and ultimately culminating in a decrease in energy dissipation. Subsequent increases in ion concentration beyond 10 μM lead to enhanced charge effects within the double layer capacitor, increasing interactions between PBS ions and the gold surface, increasing shear viscosity and subsequently energy dissipation.^[^ ^40^ ^]^ This concentration‐dependent dissipation behavior illustrates the interplay between ion adsorption, charge modulation, and viscoelastic properties at the gold–water interface.

Meanwhile, the frequency increases with the addition of ions up to ionic concentrations of 10 μM, more evident from mode 2 (Figure 2b) and mode 3 (Figure 2c), affirming that there is an addition of ionic mass on the surface (in the sensitivity range of each mode of vibration)^[^ ^58^ ^]^ upon an increase in the concentration of ions. Interestingly, the frequency does stabilize after 10 μM, indicating that the charge effect of the added ions beyond 10 μM dominates over its mass effect/contribution to the double layer capacitor. Note that no conclusions can be made from the frequency changes observed in mode 1 (Figure 2a) due to its high bulk sensitivity.

Furthermore, we also measure the bulk changes in the density (Figure 2d), viscosity (Figure 2e), and hydrodynamic mass (Figure 2f) of the phosphate buffer from concentrations ranging from 0.01 M to 1 nM. There are no significant alterations detected in the density, viscosity, and hydrodynamic mass of the bulk solution. This implies that the changes registered by the vibrating solid primarily come from the interaction of liquid layers in close proximity to the surface of the vibrating solid.

**Figure 3:**
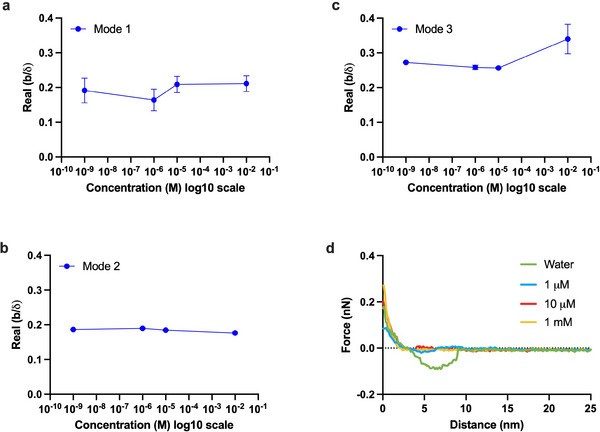
Liquid slip and ionic forces: a) depicts liquid slip occurring on the surface of the vibrating solid at 10 MHz for various ionic concentrations of PBS; b) illustrates liquid slip on the surface of the vibrating solid at 30 MHz for various ionic concentrations of PBS; c) shows liquid slip on the surface of the vibrating solid at 50 MHz for various ionic concentrations of PBS and d) depicts the force measured using AFM on the surface of the vibrating solid when exposed to water and various concentrations of PBS.

**Figure 4:**
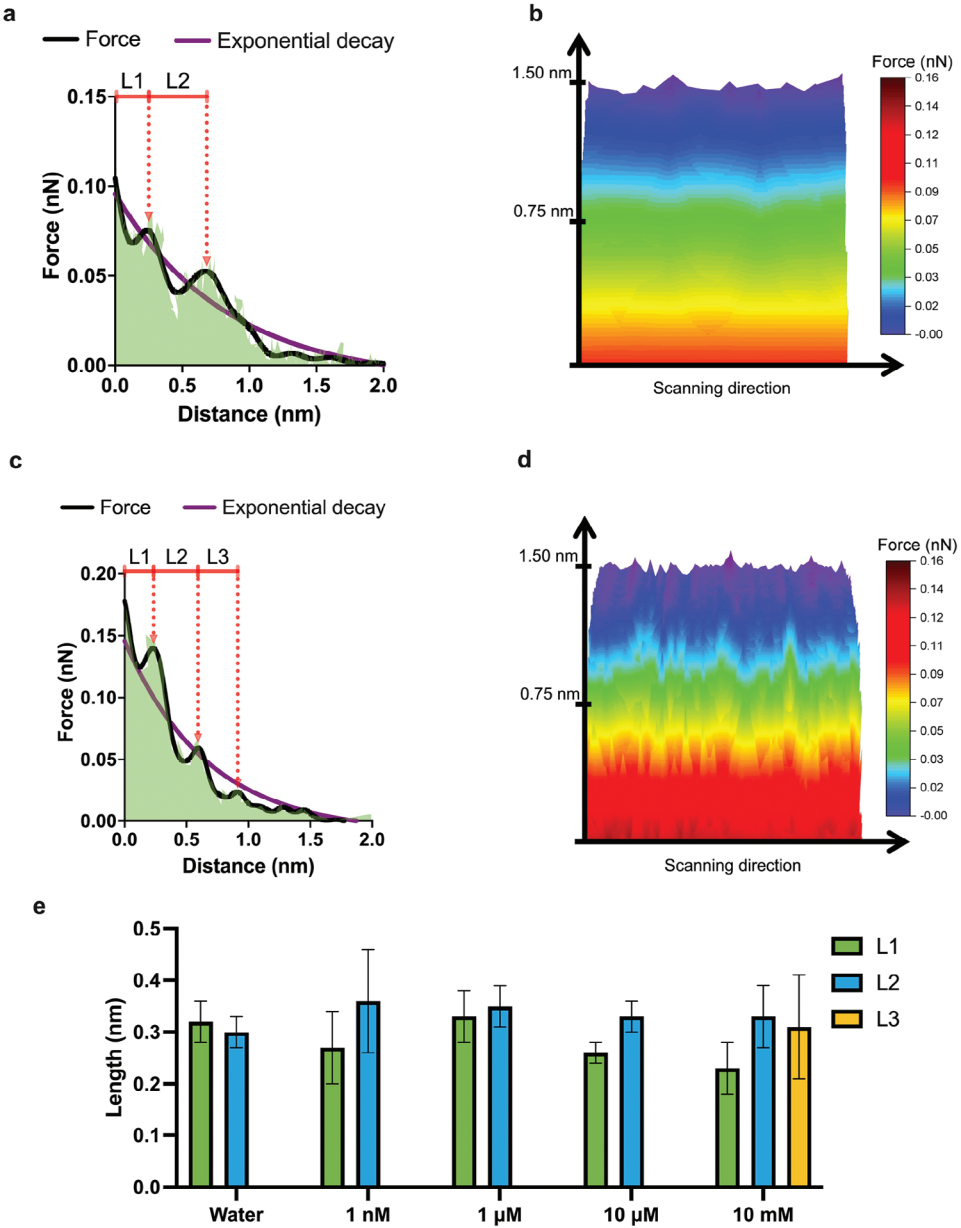
Force spectroscopy a) the forces exerted by the initial layers of deionized water on the top of a vibrating solid surface; panel (b) shows the initial layers of water; c) the forces exerted by the initial layers of 10 mM PBS, prepared in deionized water, on the upper surface of the vibrating solid; panel (d) shows the layers constituting the double‐layer capacitive structure on the upper surface of the vibrating solid, with 10 mM PBS resting on top of it; e) length of inertial layers of water and PBS on the vibrating solid surface.

Since the ions are moving inside the liquid by virtue of the thermal diffusion, moving ions have inertia that will contribute to liquid slip on the surface of the solid while ordering themselves to form the double layer capacitor. In a recent study by Greenwood et al.,^[^ ^41^ ^]^ it was found that employing graphene as a supportive non‐vibrating solid layer results in the promotion of slip due to the presence of liquid structure, such as interfacial layers of ions. This effect is observed to be equally significant as the interaction energy between the solid and liquid phases. In our case, the solid is in a vibrating state, for which the dynamic liquid slip can be explained by^[^ ^34^ ^]^

(1) $$\begin{array}{c} \frac{b}{\delta }=\left(1+i\right)\frac{\left(Z_{L}^{s}-Z_{L}^{ns}-i\omega \rho_{f}l_{a}\right)}{2(Z_{L}^{ns}+i\omega \rho_{f}l_{a})} \end{array}$$

where b/δ is a dimensionless quantity used to characterize slip length (b) with respect to penetration length (δ). Other factors in this empirical expression include load no‐slip impedance ($Z_{L}$ 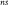), load slip impedance ($Z_{L}$ 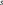), fluid density (ρ 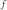), and inertial length ($l_{a}$) of the solid‐liquid interface, more details are in our previous work.^[^ ^34^ ^]^ We plot the quantity b/δ in **Figure** **3**a–c for three modes of vibrating solid, modes 1, 2, and 3, respectively. From mode 1, Figure 3a, we observe slight variations in liquid slip, primarily due to the greater penetration depth of mode 1 vibrations, approaching the bulk depth within the solution, compared to modes 2 and 3, which reside closer to the solid‐liquid interfacial depths. However, these subtle differences in mode 1 cannot be distinguished across varying concentrations of ions in the solution. Moving to the penetration depth of mode 2, we note fewer variations in slip compared to mode 1, Figure 3b, owing to the relative stability of the layers interrogated by mode 2 (when compared to mode 1). Mode 3 provides a clearer depiction of the interface compared to modes 1 and 2, as it resides closer to the interface and thus reveals more accurate interfacial effects such as slip, particularly at higher concentrations. From Figure 3c, it is evident that slip increases once ion ordering at 10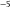 M is achieved in our study, consistent with findings in existing literature demonstrating the impact of liquid ordering on solid surfaces.^[^ ^41^ ^]^ It should be noted that to find liquid slips with the Equation (1) we have considered inertial length ($l_{a}$) between 0.3 and 0.4 nm from our experimental findings using atomic force microscopy (AFM), explained later in the text and **Figure** **4**.

To further support our observations of the formation of double layer capacitor beyond 10 μM, we perform AFM force‐distance curve (FDC) measurements. Our investigation using AFM reveals a clear evolution in force profiles with varying PBS concentrations (Figure 3d). Initially, at low concentrations (<10 μM), AFM measurements depict a balanced interaction characterized by a combination of attractive van der Waals forces and repulsive forces stemming from the EDL. This equilibrium reflects a delicate interplay where surface charge effects are mitigated by the low ionic environment. However, beyond 10 μM PBS, the AFM force profiles exhibit a pronounced shift toward predominantly repulsive forces. This shift highlights the growing influence of the EDL, which intensifies with higher ionic concentrations, leading to the formation of a more distinct EDL and consequently stronger repulsive interactions. These findings highlight the dynamic changes in surface charge and EDL dynamics as the PBS concentration increases, indicating a transition from balanced attractive and repulsive forces to a regime dominated by EDL‐mediated repulsion. This also suggests the existence of inertial effects (including slip) that ions start to contribute while forming a double layer capacitor on a solid surface.

To investigate these inertial effects, we use AFM force spectroscopy to interrogate the layers close to the surface of the solid, as shown in Figure 4. It should be noted that according to the works of Harrellson et al.^[^ ^42^ ^]^ there is an interplay of mainly 2 forces when we are within distances of less than 1 nm from the surface of a solid. These forces are 1) hydration/solvation forces and 2) forces due to chemical potential (charge related forces), which result into overall compressive stress at length scale up to 1 nm away from the surface of the solid, as also observed in other works.^[^ ^43^ ^]^ Here, using AFM, we measure the hydration/solvation force, which is an exponentially decaying force whose magnitude decreases as we move from the interface to the bulk solution, see Figure 4a for the case of water in contact with our vibrating solid sensor. The observed oscillatory‐exponential force profile in AFM force mapping reflects the interaction between the AFM tip and the hydration layers of water molecules adsorbed on the gold surface. The oscillatory component is attributed to the hydration layers, while the exponential component arises from the repulsive forces of the EDL, which become more pronounced with higher PBS concentrations.

From Figure 4a,c, we can also observe that the true nature of this hydration force (as we used deionized water) is oscillatory that is due to the tendency of the first few layers of the water molecules to order or solidify near the surface of a solid.^[^ ^44^ ^]^ This ordering of water is well‐known as the projection of inertia, which transists the liquid from a flowing to a rigid state.^[^ ^45^ ^]^ Therefore, peaks of the oscillating curve indicate the maximum force of the inertial liquid layers, and peak‐to‐peak distance indicates the length of that particular layer.

Similarly, we show how forces of these layers change when water is spiked with phosphate buffer ions. In Figure 4c, we show the forces of the layers at a PBS solution concentration of 10 mM. Interestingly, we see an additional layer, named L3, primarily due to the presence of ordered ions on the surface. These AFM force profiles confirm our findings that the structure of the EDL exhibits reconfiguration in response to changes in ion concentration. Initially, introduced ions can stabilize the EDL. Higher concentrations may lead to oversaturation, prompting additional reconfiguration and resulting in inconsistent changes in layers thickness. The interplay among electrostatic attraction, ion repulsion, and van der Waals forces can vary nonlinearly with ion concentration, thereby influencing the observed thicknesses of these layers.

We have also calculated the length of these liquid layers on the surface, which varies between 0.25 and 0.40 nm, see Figure 4b,d and e. Note that since these layers are more ordered than the bulk layers, we refer to these layers as inertial layers of the liquid on the vibrating surface. Our observation that the thickness of the layers remains within the range of 0.25–0.40 nm despite varying PBS concentrations is consistent with the understanding of hydration layer behavior at metal–water interfaces. The limited change in thickness suggests that the primary effects of PBS ion addition are on the stability and distribution of the electric double layer rather than on the physical dimensions of the hydration layers. Moreover, up to the length of L1 we can call the layer as the Helmholtz plane (consisting of the IHP and OHP) and up to the length of two layers or within the three layer distances we call the layer as the double layer capacitor. It is important to note that the double layer capacitor (up to 1 nm) differs from the Debye length, which is the charge screening length of the electrode depending on the charge of the solid. In our experiments this is better indicated by Figure 3d, where repulsive forces occurring up to few nanometers away from the surface begin to manifest on the vibrating solid.

**Figure 5:**
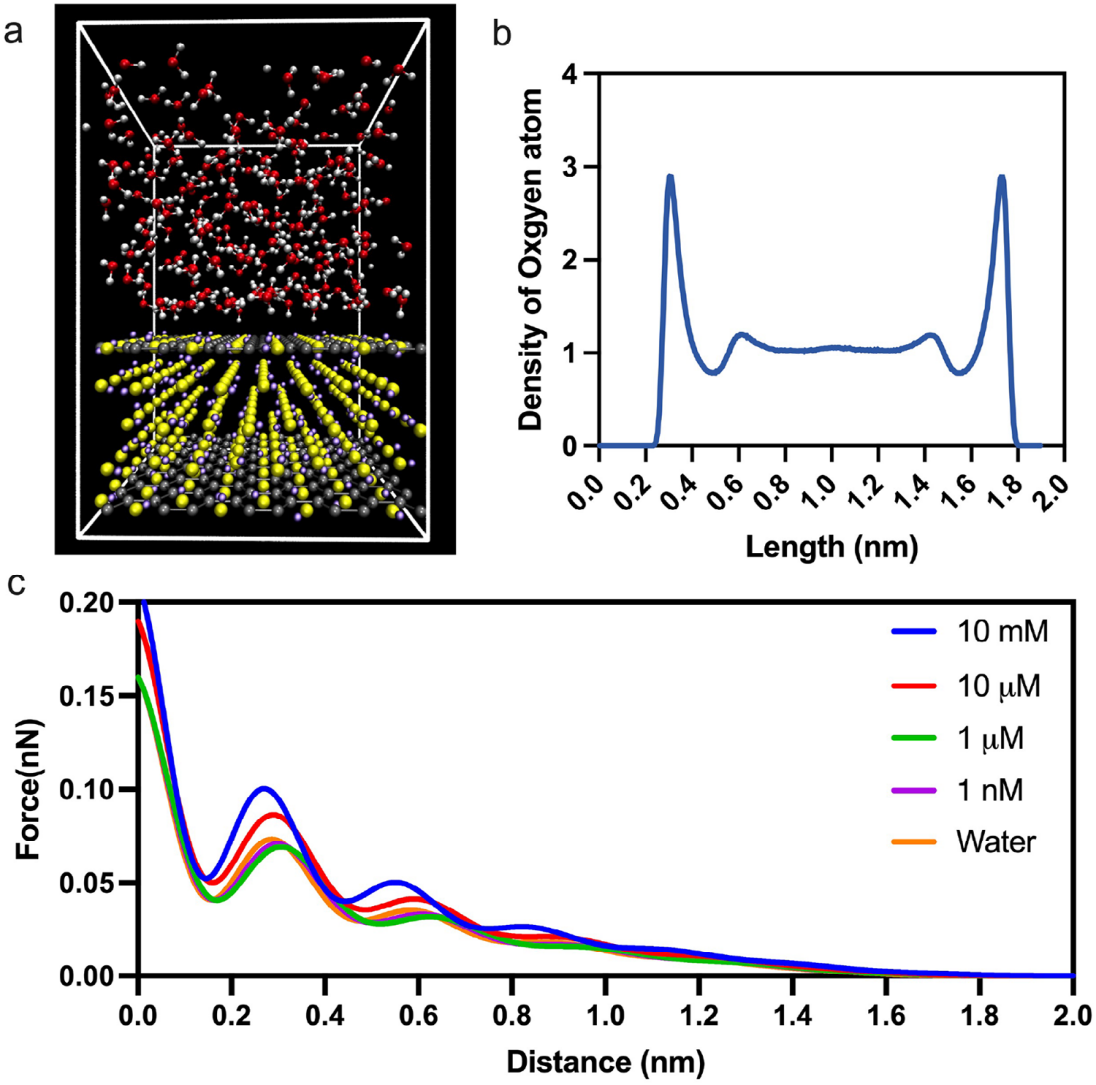
Simulations of water structure and tip‐surface interactions on gold: a) Representative snapshot of the equilibrated molecular‐dynamics system: a 2.03 × 2.05 × 1.10 nm Au(111) slab (yellow and gray spheres) is overlaid by a 1.9 nm water layer (red and white spheres), enclosed in a 2.03 × 2.05 × 3.00 nm periodic box. b) Number density profile of water‐oxygen atoms plotted as a function of distance from the Au(111) surface. Peaks at 0.308 nm correspond to the first hydration layer and the secondary maximum at 0.613 nm reflects deeper layering; the plateau between 0.4 and 1.4 nm indicates bulk‐like water behavior. Because the simulation uses periodic boundary conditions and the opposing face of the water film lies on the same Au(111) slab, a mirrored density peak appears at 1.69 nm when measured from the other side starting at 2 nm. c) Simulated total interaction force versus tip–surface separation for pure water and PBS at concentrations of 1 nM, 1 μM, 10 μM, and 10 mM.

To support our experimental observations, we also conducted molecular dynamic (MD) simulations and evaluated the results using an analytical model. The representative MD snapshot in **Figure** **5**a confirms that, after equilibration, water molecules fully wet the Au(111) surface and adopt the expected layered structure—only the first two solvation shells (highlighted by the dashed box) show significant ordering, while the bulk‐like region above appears isotropic and dynamically disordered. Furthermore, Figure 5b shows distinct oscillations in oxygen atom density within 1 nm of the Au(111) surface, which we attribute to the ordered hydration layer. Essentially, the first hydration layer peaks at 0.308 nm, in excellent agreement with AFM measurements of water‐adsorbed gold surfaces, which report a 0.3 nm thickness for the initial water monolayer. A secondary layering peak at 0.613 nm indicates registry of the second hydration shell.

Beyond ∼0.8 nm, the density plateaus near unity, signifying the onset of bulk‐like water structure. The symmetric peak at 1.692 nm arises from the periodic‐boundary condition, whereby water molecules on the opposite side of the slab replicate the same layering pattern. These oscillatory features confirm that molecular ordering of water persists over two to three solvation layers at ambient temperature under the chosen GolP–TIP3P force‐field parameters. Figure 5c illustrates the analytical evaluation of the total interaction force as a function of tip–surface separation for pure water and PBS at concentrations spanning five orders of magnitude using Equation (2). The total interaction force *F_int_* (D) at distance D is modelled as:

(2) $$\begin{array}{ccc} F_{int\;}\left(D\right)\; & = & \;A_{exp}\;e^{-D/\lambda_{exp}}\;+\;A_{osc}\;\cos\left(\frac{2\pi D}{d_{layer}}\right)e^{-\frac{D}{\lambda_{osc}}}\\ & & +\;F_{vdW}\;+\;F_{EDL} \end{array}$$

here, $A_{exp}$ and λ 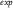 represent the amplitude and decay length of the monotonic solvation component, while $A_{osc}$, λ 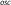, and $d_{layer}$ define the oscillatory part associated with hydration or ionic layering. The vdW term is calculated using the Hamaker constant for the gold–liquid interface and accounts for geometric smoothing to avoid divergence at small distances. The EDL force is incorporated as a repulsive exponential decay scaled by the Debye length, which varies inversely with the square root of the ionic concentration.

All curves exhibit pronounced oscillatory solvation forces at separations < 1 nm, reflecting the discrete expulsion of successive hydration layers. In pure water (orange), the monotonic decay beyond the oscillations is weak, as no electrostatic double layer (EDL) force is present. Addition of PBS introduces a long‐range, exponentially decaying repulsion whose decay length (Debye length) contracts with rising ionic strength. Furthermore, the amplitude of the short‐range oscillations increases modestly with salt concentration, consistent with slight dielectric screening effects on the effective Hamaker constant and enhanced ordering of interfacial ions. By decomposing the total force into solvation, van der Waals, and EDL components (not shown), we confirm that the solvation force dominates at < 1 nm, van der Waals contributes an attractive background at intermediate separations, and EDL repulsion governs the long‐range tail in ionic solutions. These results demonstrate how ionic strength modulates both the extent and strength of interfacial forces relevant to AFM measurements at nanometer separations.

## 3 Conclusions

In this work, we have demonstrated that 1) a mechanically ordered, sub‐nanometer “inertial” liquid layer adjacent to gold dominates energy dissipation at low ionic strength; 2) this layer recruits counterions into a tunable Inner/Outer Helmholtz plane whose slip impedance scale systematically with solution charge; and 3) beyond the classical Debye length (≈1–5 nm), mass‐loading and shear‐viscosity effects decouple charge screening from the diffuse sheath, giving rise to nonlinear QCM frequency shifts and enhanced dissipation. Using QCM and AFM force spectroscopy across PBS concentrations from 1 nM to 10 mM, we directly mapped how ion stabilization at low concentration reduces slip (balancing van der Waals attraction and EDL repulsion), while higher ionic strength amplifies viscoelastic response and drives a predominantly repulsive regime with clear oscillatory‐exponential hydration profiles. Although these findings reconcile aspects of Gouy–Chapman–Stern models with molecular‐scale mechanics, alternative interpretations—such as multilayer ion adsorption or specific ion–surface chemistries—cannot yet be fully excluded. Future studies combining in situ vibrational spectroscopy and more detailed molecular dynamics will be critical to validate the mechanistic links between impedance changes, solvation structure, and charge screening. Altogether, our results furnish a quantitative, layer‐by‐layer picture of interfacial inertia, slip and screening that can guide the predictive engineering of nanofluidic devices, catalytic interfaces, and biosensing platforms.

## 4 Experimental Section

AT‐cut piezoelectric quartz crystals, with gold electodes for electrical connectivity, were purchased from Open QCM, Italy, featuring a 10 MHz vibration frequency (AT10‐14‐6‐AU‐WRAP). Phosphate‐buffered saline (PBS) (P4417, Sigma‐Aldrich, United Kingdom) was used to prepare a water‐based salt solution. Dissolving one tablet in 200 mL of deionized water yields concentrations of 10 mM phosphate buffer, 2.7 mM potassium chloride, and 137 mM sodium chloride. By diluting a 10 mM PBS solution with deionized water, PBS solutions spanning concentrations from 1 mM down to 1 nM were prepared, encompassing a range of 1 mM, 100 μM, 10 μM, 1 μM, 100 nM, 10 nM, and 1 nM. Please note that this refers to concentration of phosphate ions in. the PBS.

### QCM Measurements

The quartz crystals were mounted on the pipetting module for QCM‐D (openQCM NEXT, Open QCM, Italy), equipped with dissipation monitoring, simultaneous multiple overtones measurement, and active thermal control capabilities. Subsequently, 0.1 mL of either deionized water or PBS solutions of varied concentrations was pipetted following a 10‐min stability period. All measurements were conducted over a 15‐min duration (excluding the stability period), with the temperature held constant at 25 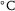. Please note that the drive voltage of the QCM is 400 mV, which would yield mechanical vibration of around 3–3.5 nm.^[^ ^46^ ^]^

### AFM Measurements

The atomic force microscope (AFM) (Jupiter XR AFM, OXFORD Instruments Asylum Research, United States of America) was used to obtain the force and interfacial layer structures. Furthermore, the AFM system was used to measure the frequency and quality factor of cantilevers in bulk water with different concentrations of PBS in order to measure the density and viscosity of liquid.^[^ ^47^ ^]^ Moreover, in AFM measurements, force‐distance curve^[^ ^48^, ^49^ ^]^ and force mapping^[^ ^50^, ^51^, ^52^ ^]^ were used to recover the force versus distance profile as well as the interfacial structure of the gold–water interface at different PBS concentrations. All measurements used cantilevers with a spring constant of k ≈ 3 N m^−1^ (RFESOA‐75, Bruker, United States of America) and PPP‐NCHAuD‐10 with a spring constant of k ≈ 42 N m^−1^.

### Molecular Dynamic Simulations

Gold protein (GolP) force field^[^ ^53^ ^]^ was used to describe the Au‐Au and Au‐water interactions. The Transferable Intermolecular Potential with 3 Points (TIP3P) water model^[^ ^54^ ^]^ was used for compatibility with the GolP parametrization. A 2.03 × 2.05 × 1.10 nm gold slab was enclosed in a simulation box of 2.03 × 2.05 × 3.00 nm. The gold slab comprised 280 Au atoms (112 on the surface and 168 in the bulk), 224 Leonard Jones (LJ) virtual sites that occupied the hollow site on the surface, and 280 LJ virtual sites for the point charges. One hundred ninety‐five water molecules were used to make density 1.0 as same as the bulk water. The system was subjected to steepest‐descent energy minimization in order to remove clashes between the atoms and ensure starting from a reasonable structure. After the energy was minimized, a 500 ps isothermal NVT simulations (number of particles, volume, and temperature constant) with a Nosé–Hoover thermostat at 300 K was performed for the initial equilibration run. Afterward, a 10 ns MD production was conducted in the same NVT ensemble (time step of 1 fs) at 300 K using a Nosé−Hoover thermostat.^[^ ^55^ ^]^ During these steps, Particle Mesh Ewald electrostatic summation was cut off at 0.8 nm, and a force‐switched cut off starting at 0.7 nm and ending at 0.8 nm was used for LJ non‐bonded interactions. Periodic boundary conditions were adopted in all simulations. The MD simulations were all performed by GROMACS 2022.4.^[^ ^56^ ^]^

### Data Analysis

In order to measure density, viscosity, and hydrodynamic mass of the bulk liquids, the first, second, and third eigenfrequencies of cantilevers and their quality factors were collected using thermal methods at water and different concentrations of PBS. Previously developed methods^[^ ^47^ ^]^ were applied to calculate simultaneously density, viscosity, and hydrodynamic mass at different PBS concentrations. Furthermore, in AFM‐FDC and force mapping analysis, the amplitude and phase versus distance curves measured by AC blue drive tapping mode AFM were recorded, and the force reconstruction methods were applied to recover the force profiles^[^ ^48^, ^50^, ^57^ ^]^ and maps of interfacial layers. Computation related to modeling AFM‐tip interaction with gold/water/PBS was conducted using MATLAB.

## Conflict of Interest

The authors declare no conflict of interest.

## Author Contributions

N.B. and Y.S. are joint first authors as Y.S. contributed to most of the experiments. N.B., D.L., and A.F.P. concieved the idea, conducted analysis, and prepared the draft of this manuscipt. J.Y. did molecular dynamics simulations. All authors revised and edited the manuscript.

## Supporting information

Supporting Information

## Data Availability

The data that support the findings of this study are available from the corresponding author upon reasonable request.

## References

[1] I. Langmuir , Chem. Rev. 1930, 6, 451.

[2] J. Yin , X. Li , J. Yu , Z. Zhang , J. Zhou , W. Guo , Nat. Nanotechnol. 2014, 9, 378. 24705513 10.1038/nnano.2014.56

[3] R. Futamura , T. Iiyama , Y. Takasaki , Y. Gogotsi , M. J. Biggs , M. Salanne , J. Ségalini , P. Simon , K. Kaneko , Nat. Mater. 2017, 16, 1225. 28920938 10.1038/nmat4974 PMC5702543

[4] M. A. Gebbie , A. M. Smith , H. A. Dobbs , G. G. Warr , X. Banquy , M. Valtiner , M. W. Rutland , J. N. Israelachvili , S. Perkin , R. Atkin , Chem. Commun. 2017, 53, 1214. 10.1039/c6cc08820a 28000809

[5] K. R. Siefermann , Y. Liu , E. Lugovoy , O. Link , M. Faubel , U. Buck , B. Winter , B. Abel , Nat. Chem. 2010, 2, 274. 21124507 10.1038/nchem.580

[6] A. Verdaguer , G. M. Sacha , H. Bluhm , M. Salmeron , Chem. Rev. 2006, 106, 1478. 16608188 10.1021/cr040376l

[7] M. A. Brown , A. Goel , Z. Abbas , Angew. Chem. 2016, 128, 3854. 10.1002/anie.201512025 26880184

[8] W. Trewby , M. Tavakol , Y. M. Jaques , K. Voitchovsky , Mater. Today Phys. 2024, 101441.

[9] M. Favaro , B. Jeong , P. N. Ross , J. Yano , Z. Hussain , Z. Liu , E. J. Crumlin , Nat. Commun. 2016, 7, 12695. 27576762 10.1038/ncomms12695 PMC5013669

[10] S. Sarkar , M. J. Gukeh , T. Roy , H. Gaikwad , F. M. Bellussi , S. Moitra , C. M. Megaridis , J. Colloid Interface Sci. 2023, 633, 800. 36493744 10.1016/j.jcis.2022.10.101

[11] C. Sievers , Y. Noda , L. Qi , E. M. Albuquerque , R. M. Rioux , S. L. Scott , ACS Catal. 2016, 6, 8286.

[12] S. Zhang , Y. Hou , L. Zhang , H. Zhu , J. Xiong , S. Wang , T. Liu , Small 2024, 20, 2311816. 10.1002/smll.202311816 38396322

[13] F. Zaera , Chem. Rev. 2012, 112, 2920. 22277079 10.1021/cr2002068

[14] G. G. Geesey , D. C. White , Annu. Rev. Microbiol. 1990, 44, 579. 2252395 10.1146/annurev.mi.44.100190.003051

[15] S. Lin , X. Chen , Z. L. Wang , Chem. Rev. 2021, 122, 5209. 34160191 10.1021/acs.chemrev.1c00176

[16] J. A. Lewis , F. J. Q. Cortes , Y. Liu , J. C. Miers , A. Verma , B. S. Vishnugopi , J. Tippens , D. Prakash , T. S. Marchese , S. Y. Han , Nat. Mater. 2021, 20, 503. 33510445 10.1038/s41563-020-00903-2

[17] H. Su , W. Zhou , H. Zhang , W. Zhou , X. Zhao , Y. Li , M. Liu , W. Cheng , Q. Liu , J. Am. Chem. Soc. 2020, 142, 12306. 32579351 10.1021/jacs.0c04231

[18] X. Su , Z. Wan , Y. Lu , O. Rojas , Langmuir 2024, 40, 4881. 38386001 10.1021/acs.langmuir.3c03787

[19] N. Ubrig , E. Ponomarev , J. Zultak , D. Domaretskiy , V. Zólyomi , D. Terry , J. Howarth , I. Gutiérrez‐Lezama , A. Zhukov , Z. R. Kudrynskyi , et al., Nat. Mater. 2020, 19, 299. 32015532 10.1038/s41563-019-0601-3

[20] H. Liu , L. Jiang , B. Cao , H. Du , H. Lu , Y. Ma , H. Wang , H. Guo , Q. Huang , B. Xu , et al., ACS Nano 2022, 16, 14539. 36067370 10.1021/acsnano.2c04968

[21] M. P. Ruiz , J. A. Faria , ACS Eng. Au 2022, 2, 295.

[22] D. S. Potts , D. T. Bregante , J. S. Adams , C. Torres , D. W. Flaherty , Chem. Soc. Rev. 2021, 50, 12308. 34569580 10.1039/d1cs00539a

[23] S. A. Pervez , G. Kim , B. P. Vinayan , M. A. Cambaz , M. Kuenzel , M. Hekmatfar , M. Fichtner , S. Passerini , Small 2020, 16, 2000279. 10.1002/smll.202000279 32105407

[24] C. Yin , J. Li , T. Li , Y. Yu , Y. Kong , P. Gao , H. Peng , L. Tong , J. Zhang , Adv. Funct. Mater. 2020, 30, 2001396.

[25] Y. Chen , W. Wu , S. Gonzalez‐Munoz , L. Forcieri , C. Wells , S. P. Jarvis , F. Wu , R. Young , A. Dey , M. Isaacs , Nat. Commun. 2023, 14, 1321. 36898996 10.1038/s41467-023-37033-7 PMC10006426

[26] X. Peng , F.‐C. Zhu , Y.‐H. Jiang , J.‐J. Sun , L.‐P. Xiao , S. Zhou , K. C. Bustillo , L.‐H. Lin , J. Cheng , J.‐F. Li , et al., Nat. Commun. 2022, 13, 3601. 35739085 10.1038/s41467-022-31075-z PMC9226024

[27] Q. Sun , B. Ge , B. Xiao , F. Li , L. Ji , Z. Yin , J. Guo , J. Tang , C. Zhou , W. Jie , Adv. Sci. 2023, 10, 2302236. 10.1002/advs.202302236 PMC10427374 37282775

[28] L. Zhang , C. Huang , L. Yang , Y. Zhou , S. Yao , K. Sun , N. Geng , G. Huang , L. Liang , Y. Xie , Surf. Interfaces 2024, 48, 104327.

[29] O. Björneholm , M. H. Hansen , A. Hodgson , L.‐M. Liu , D. T. Limmer , A. Michaelides , P. Pedevilla , J. Rossmeisl , H. Shen , G. Tocci , et al., Chem. Rev. 2016, 116, 7698. 27232062 10.1021/acs.chemrev.6b00045

[30] L. Bellarosa , R. García‐Muelas , G. Revilla‐López , N. López , ACS Cent. Sci. 2016, 2, 109. 26937488 10.1021/acscentsci.5b00349 PMC4768339

[31] A. Hodgson , S. Haq , Surf. Sci. Rep. 2009, 64, 381.

[32] J. C. Palmer , F. Martelli , Y. Liu , R. Car , A. Z. Panagiotopoulos , P. G. Debenedetti , Nature 2014, 510, 385. 24943954 10.1038/nature13405

[33] F. Tang , T. Ohto , S. Sun , J. R. Rouxel , S. Imoto , E. H. Backus , S. Mukamel , M. Bonn , Y. Nagata , Chem. Rev. 2020, 120, 3633. 32141737 10.1021/acs.chemrev.9b00512 PMC7181271

[34] A. F. Payam , B. Kim , D. Lee , N. Bhalla , Nat. Commun. 2022, 13, 6608. 36329039 10.1038/s41467-022-34319-0 PMC9633805

[35] T. K. Lind , M. Cárdenas , Biointerphases 2016, 11. 10.1116/1.4944830 27033712

[36] J. Hu , G. Yesilbas , Y. Li , X. Geng , J. Chen , X. Wu , A. Knoll , T.‐L. Ren , Anal. Chem. 2023, 95, 4043. 36800209 10.1021/acs.analchem.2c04510

[37] F. Tang , T. Ohto , S. Sun , J. R. Rouxel , S. Imoto , E. H. Backus , S. Mukamel , M. Bonn , Y. Nagata , Chem. Rev. 2020, 120, 3633. 32141737 10.1021/acs.chemrev.9b00512 PMC7181271

[38] I. L. Geada , H. Ramezani‐Dakhel , T. Jamil , M. Sulpizi , H. Heinz , Nat. Commun. 2018, 9, 716. 29459638 10.1038/s41467-018-03137-8 PMC5818522

[39] L. Xiang , P. Zhang , C. Liu , X. He , H. B. Li , Y. Li , Z. Wang , J. Hihath , S. H. Kim , D. N. Beratan , Matter 2020, 3, 166. 33103114 10.1016/j.matt.2020.03.023 PMC7584381

[40] R. Funari , A. Matsumoto , J. R. D. Bruyn , A. Q. Shen , Anal. Chem. 2020, 92, 8244. 32419462 10.1021/acs.analchem.0c00475

[41] G. Greenwood , J. M. Kim , Q. Zheng , S. M. Nahid , S. Nam , R. M. Espinosa‐Marzal , ACS Nano 2021, 15, 10095. 34114798 10.1021/acsnano.1c01884

[42] S. G. Harrellson , M. S. DeLay , X. Chen , A.‐H. Cavusoglu , J. Dworkin , H. A. Stone , O. Sahin , Nature 2023, 1. 10.1038/s41586-023-06144-y PMC10530534 37286609

[43] J.‐J. Velasco‐Velez , C. H. Wu , T. A. Pascal , L. F. Wan , J. Guo , D. Prendergast , M. Salmeron , Science 2014, 346, 831. 25342657 10.1126/science.1259437

[44] S. H. Khan , G. Matei , S. Patil , P. M. Hoffmann , Phys. Rev. Lett. 2010, 105, 106101. 20867530 10.1103/PhysRevLett.105.106101

[45] A. J. Liu , S. R. Nagel , Annu. Rev. Condens. Matter Phys. 2010, 1, 347.

[46] M. Ishikawa , N. Wada , T. Miyakawa , H. Matsukawa , M. Suzuki , N. Sasaki , K. Miura , Physical Review B 2016, 93, 201401.

[47] A. F. Payam , W. Trewby , K. Voitchovsky , Analyst 2017, 142, 1492. 28352874 10.1039/c6an02674e PMC5450008

[48] A. F. Payam , D. Martin‐Jimenez , R. Garcia , Nanotechnology 2015, 26, 185706. 25876817 10.1088/0957-4484/26/18/185706

[49] A. F. Payam , R. Funari , G. Scamarcio , N. Bhalla , Small Sci. 2023, 3, 2300029. 40213609 10.1002/smsc.202300029 PMC11935963

[50] D. Martin‐Jimenez , E. Chacon , P. Tarazona , R. Garcia , Nat. Commun. 2016, 7, 12164. 27416784 10.1038/ncomms12164 PMC4947176

[51] L. K. S. Bonagiri , K. S. Panse , S. Zhou , H. Wu , N. R. Aluru , Y. Zhang , ACS Nano 2022, 16, 19594. 36351178 10.1021/acsnano.2c10819

[52] M. R. Uhlig , D. Martin‐Jimenez , R. Garcia , Nat. Commun. 2019, 10, 2606. 31197159 10.1038/s41467-019-10740-w PMC6565678

[53] F. Iori , R. D. Felice , E. Molinari , S. Corni , J. Comput. Chem. 2009, 30, 1465. 19037859 10.1002/jcc.21165

[54] W. L. Jorgensen , J. Chandrasekhar , J. D. Madura , R. W. Impey , M. L. Klein , J. Chem. Phys. 1983, 79, 926.

[55] S. Nosé , M. L. Klein , Mol. Phys. 1983, 50, 1055.

[56] M. J. Abraham , T. Murtola , R. Schulz , S. Páll , J. C. Smith , B. Hess , E. Lindahl , SoftwareX 2015, 1, 19.

[57] H. Hölscher , Appl. Phys. Lett. 2006, 89.

[58] S. Jana , N. Bhalla , Adv. Func. Mater. 2024, 35, 10.1002/adfm.20241468.

