# Supplementary material for: Resolving Nanoslip, Solvation Inertia, and Charge Dynamics at Vibrating Solid–Liquid Interface
Source: Small. 2025 Jul 31;21(35):2505067. doi: 10.1002/smll.202505067 (PMC12410916; doi:10.1002/smll.202505067)
Supplement: Supplementary file 1 — Supporting Information [file SMLL-21-2505067-s001.docx]

**Supplementary Information**

**Resolving nanoslip, solvation inertia and charge dynamics at vibrating solid-liquid interface**

*Nikhil Bhalla^1*%^, Yeeun Song^2%^, Ju-Yeon Jo^3^, Doojin Lee^2*^ & Amir Farokh Payam^1*^*

*^1^Nanotechnology and Integrated Bioengineering Centre (NIBEC), School of Engineering, Ulster University, 2-24 York Street, Belfast, Northern Ireland BT15 1AP, United Kingdom*

*^2^School of Polymer Science and Engineering & Department of Polymer Engineering, Graduate School, Chonnam National University, 77 Yongbong-ro, Buk-gu, Gwangju, 61186, Republic of Korea*

*^3^Graduate School of Energy Science, Kyoto University, Yoshida-Honmachi, Sakyo-ku, Kyoto 606-8501, Japan*

**Corresponding authors*

*% NB and YS are joint first authors*

Table of Contents

[1. Thermal tuning measurements of AFM cantilever 3](#_Toc196427442)

# **Thermal tuning measurements of AFM cantilever**

We analyse the frequency response of a free cantilever at three frequencies, corresponding to its eigenmodes. We also determine the quality factor. This experiment was done to examine the impact of frequency variation on the calculation of density and viscosity, shared in the manuscript text (Figure 2). Detailed results are presented in Figures S1.

***Figure S1:*** *a) Frequency response of eigenmode 1. b) Quality factor of eigenmode 1. c) Frequency response of eigenmode 2. d) Quality factor of eigenmode 2. e) Frequency response of eigenmode 3. f) Quality factor of eigenmode 3.*
